# Goal setting and knowledge generation through health policy and systems research in low- and middle-income countries

**DOI:** 10.1186/1478-4505-12-39

**Published:** 2014-08-14

**Authors:** Jiayin Xue, Bhavini Murthy, Nhan T Tran, Abdul Ghaffar

**Affiliations:** 1 Department of Pediatrics, Massachusetts General Hospital, 5th Floor, 175 Cambridge St, Boston, MA 02114, USA; 2 Department of Social Medicine, UNC School of Medicine, 333 South Columbia Street, MacNider Hall, Rm#348/ CB#7240, Chapel Hill, NC 27599, USA; 3 Alliance for Health Policy & Systems Research, World Health Organization, Health Systems and Innovation Cluster, 20 Avenue Appia, 1211 Geneva, Switzerland

**Keywords:** Country cooperation strategies, Health policy and systems research, Low- and middle-income countries, World Health Organization

## Abstract

The importance of health policy and systems research (HPSR) and its role in aiding health system reforms has been increasingly recognized in recent years within the World Health Organization (WHO). An assessment of the 71 WHO Country Cooperation Strategies (CCS) that are publicly available and were published in English in 2012 was completed to determine the extent to which HPSR goals are incorporated at the global level. A review was then conducted using a Medline database search to determine the number of articles published by countries with HPSR goals. Sixty-six out of the 71 (93%) available CCS describe HPSR as an objective or strategy for achieving health system priorities. However, only 52 out of the 66 countries (79%) have any publications involving HPSR during their most recent CCS cycle. This suggests that although health systems strengthening through HPSR is increasingly emphasized by the WHO and country health ministries, actual HPSR progress may still be lacking. There is a need and an opportunity for the WHO and other global health agencies to focus on providing the necessary tools and building HPSR capacity in low- and middle-income countries.

## Background

Despite recent advances in medicine and technology, many countries still struggle to build effective health systems that can successfully deliver life enhancing interventions. Researchers are increasingly utilizing health policy and systems research (HPSR) as a means of generating evidence to improve health systems. HPSR is a multidisciplinary field that encompasses research in one or more of the health system building blocks as defined by the World Health Organization (WHO), namely leadership and governance, health financing, health workforce, health services, medical products, vaccines and technologies, and health information [1,2].

The WHO, in releasing its 2012 Strategy on HPSR Changing Mindsets, emphasized the value and role of HPSR in strengthening health systems to meet global and national health goals [3]. To assist Member States in this matter, the WHO, in collaboration with the national teams, developed a set of Country Cooperation Strategies (CCS) that are used to guide the work undertaken by many low- and middle-income countries (LMICs). In many of the CCS, conducting research in one or more of the health system building blocks is considered a strategy for achieving health system priorities or as a research goal for the country.

In an attempt to assess the extent to which HPSR has been considered by WHO at the global level and country levels, available CCS were analyzed to see which goals and actions, if any, relate to HPSR. In order to understand how these goals affect research at the country level, a Medline database search was conducted to determine if there is a correlation between the volume of HPSR literature published in countries where HPSR goals are clearly articulated in the CCS, assuming that peer-reviewed publications are reflective of the amount of research activity undertaken.

## HPSR goal setting through country cooperation strategies

In 1998, the WHO recommended the development of country-specific strategies to improve coordination between health sector partners [4]. In the following year, the structure of a CCS was agreed upon by the WHO and the Member States to outline each country’s medium and long term health priorities during a 4 to 6 year cycle [4]. According to the 2010 WHO Country Presence Report, 145 WHO country offices had a CCS, and 85% of them used it to guide the country’s planning and operations in advocacy, resource procurement, management, and technical support. Only 71 of the most current CCS were publicly available and published in English at the time of our review [5]. The extent and the types of HPSR mentioned in the CCS were assessed based on the health system building blocks involved. Clinical and basic science research and monitoring and evaluation of program progress were not considered HPSR [2].

**Figure 1:**
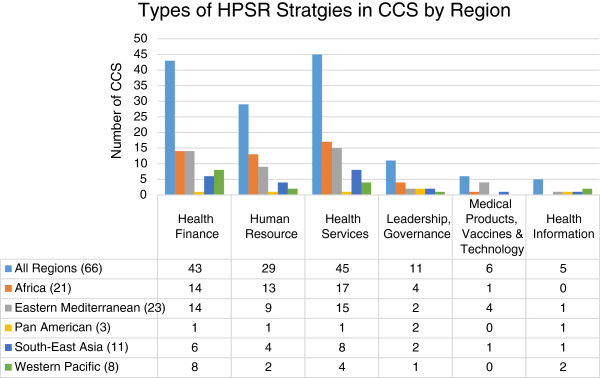
**Variations on the types of research on health system building blocks as a part of CCS objectives and strategies.** (#) indicate the total number of CCS with HPSR emphasis from each region.

The 71 CCS represent 22 countries from the African region, 23 from the Eastern Mediterranean region, 6 from the Americas, 11 from the South-East Asia region, and 9 from the Western Pacific region. All but 5 of the CCS describe research in one of the health policy and systems building blocks as a strategy for achieving health systems strengthening and development. Given that the CCS is only a blueprint for policy makers, there are no specific guidelines or standards on how much should be invested in HPSR and how this work should be undertaken.Of the 71 CCS reviewed, 66 included some goals or objectives that related to the use of HPSR. The majority of these include some emphasis on knowledge generation and the use of research evidence in policy and other decision making. About one third of the CCS mention health systems and implementation research and capacity building. Among the six health systems building blocks, goals and objectives related to health finance and service delivery are addressed in nearly two thirds of the strategies. Research and analysis of human resources are included in over 40% of the CCS, research on governance and management are included in over 16%, and research on cost-effectiveness analysis for medical technologies and the health information system are mentioned in less than 10% (Figure 1). Although health information technology (HIT) research is not emphasized, in most of the CCS, there was acknowledgement of the importance of strengthening HIT in general.

### Published HPSR

A Medline review of HPSR articles originating from each of the 66 countries with CCS priorities in HPSR was conducted. The search strategy for the analysis included articles under the categories of “Health Care Economics and Organizations”, “Health Care Facilities, Manpower, and Services”, “Health Care Quality, Access, and Evaluation”, and “Health Services Administration/Economics”.^a^ These were chosen after reviewing all of Medline’s health-related headings, and all of the aforementioned health systems building blocks are included under one or more of these overarching categories, and the same literature search was performed for all countries of interest. Due to variability in the duration and timing of the CCS cycles, it was not possible to evaluate all HPSR publications during a standard time frame for all countries. Instead, titles and abstracts of articles published during the span of the most recent CCS cycle or up to December 31, 2012 were reviewed.

**Figure 2:**
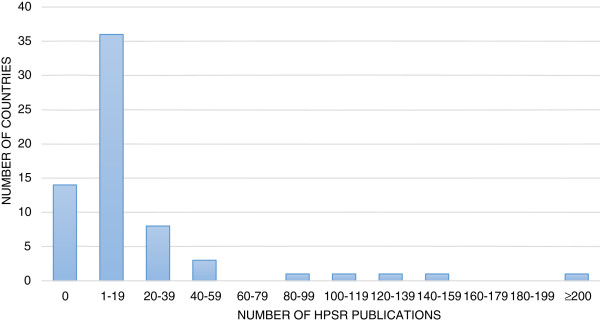
Histogram of the number of HPSR publications by countries that incorporated HPSR goals into the most recent CCS cycles.

Clinical and basic science research articles were excluded unless they had direct implications for health policy or cost-effectiveness. Publications were limited to those generated from each country of interest rather than work done for LMICs by foreign institutes and researchers. To minimize bias, two reviewers independently reviewed all the CCS and Medline articles for adherence to our definition of HPSR. Any discordancy was discussed and the final number of publications included was agreed upon by both reviewers.Using our search criteria, 14 out of the 66 (21%) countries did not have any HPSR publications during their most recent CCS cycle. African countries made up the largest portion of those without any publications (6 out of 14). Of those that had any HPSR publications, 36 countries had less than 20 publications, 10 countries had 20 to 50 publications, and 6 countries had 50 or more publications (Figure 2). The countries that had more than 50 publications in HPSR were Kenya, South Africa, Nigeria, China, South Pacific countries, and Brazil. However, all but one publication from the South Pacific countries came from New Zealand. Of note, India also had more than 50 publications from its previous CCS cycle, but the current cycle started in 2012, and therefore our review only included publications from January to December, 2012.

## Discussion

The use of HPSR evidence for improved performance of health systems is increasingly emphasized by WHO as evidenced by its inclusion in the CCS. However, upon closer examination of the CCS, HPSR goals are often buried among numerous other strategies for achieving health systems strengthening, and very few CCS have specific guidance on how to carry out these strategies. Many country priorities seem wide in scope and echo regional priorities rather than focusing on country-specific issues.

Despite the prevalence of HPSR contents in the CCS, actual progress in the number of publications by country is still less than desirable with over one fifth of the countries having no HPSR publications during their respective CCS cycle. While there are aspects of HPSR that are explicitly listed in the CCS, there may still be a lack of attention and interest among researchers and implementers, especially given other competing priorities such as basic science and clinical research, technology development, prevention of communicable and non-communicable diseases, etc.

Among the countries with the highest number of HPSR publications, New Zealand is a high income country, and South Africa, China, and Brazil are considered upper-middle income countries according to the World Bank’s gross national income per capita classifications. This reflects the limited HPSR capacity in many LMICs due to both shortages of trained research personnel and local funding for HPSR [6]. There are many researchers from North American and Western European countries who are actively involved in HPSR for LMICs; their work is not captured in this review since we wanted to assess the ability of LMICs to independently generate research.

Currently, most of the funding for HPSR comes from donor organizations such as the UK Department for International Development (DFID), the Norwegian Government Agency for Development Cooperation (NORAD), the African Institute for Development Policy (AFIDEP), the Wellcome Trust, and the WHO. As stated in the 2004 Ministerial Summit on Health Research and the WHO Strategy on Research for Health, LMIC countries are expected to invest at least 2% of national health expenditures and 5% of healthcare aids in research and research capacity building and “*to allocate adequate funding and human resources for health systems research*” [7,8].

The CCS is an agreement between the WHO and the countries that it supports. It is the responsibility of both the WHO and the country to identify areas where it falls short and to strategize ways to better allocate resources. On the country level, improved health research system infrastructure and inclusion of agendas and budgets that reflect HPSR goals can better facilitate the conduct and use of HPSR. As a global actor, WHO and its hosted entities can help monitor the field of HPSR as well as provide guidance on how to build capacity for HPSR and how to support its use in the context of health systems decision making.

### Limitations

The definition of HPSR based on health systems building blocks may not include all facets of HPSR. However, we used this definition to be consistent with previous work in this field [2]. Our review may not reflect the scope of HPSR priorities expressed in all CCS reports, since only 71 CCS reports were publicly available in English. The analysis was also confined to Medline/PubMed, which may not have indexed all foreign language journals, although our search strategy was not limited to the English language. In order to fully summarize the extent of health systems research in LMICs, non-published reports and other work taken by major research organizations and governments should also be examined.

The current comparison uses publications within the same time frame as the CCS documents reviewed, but more time may be needed between goal setting and research publications to accurately assess the amount of research conducted in these countries. However, because the span of the CCS cycles varied by country, it was not possible to standardize the search with a lag time given that some cycles were just beginning while others had ended at the time of the literature search.

Because of the large number of countries and articles involved, we did not attempt to further delineate the relationship between the types of HPSR published and the goals that were stated in each individual CCS. A more in-depth analysis may be useful in clarifying the types of studies published and to determine whether these HPSR publications are regarding new interventions or the scaling up of existing programs. Furthermore, surveys of country health budgets would provide useful information on how much funding is actually allocated to HPSR at the country level.

Our analysis also did not investigate whether evidence generation directly resulted in the translation and dissemination of knowledge. Additional studies should be conducted to determine whether HPSR is being effectively used by governments and institutions in LMICs to improve health systems.

## Conclusions

There is a growing recognition of the value of HPSR in facilitating health systems improvement and health outcomes; its role in improving policy making has been recognized by the WHO and the Member States [3]. However, evidence of research progress still appears to be limited for many LMICs based on the volume of publications. Inclusion of HPSR in the CCS is a crucial first step, but inclusion does not imply that there are capacities in place to generate and to use policy-relevant knowledge. There is an opportunity for WHO Headquarters and other global actors to increase support in this area, for there may not have been adequate tools, funding, and capacity provided for the countries to meet their HPSR goals. Coordinated efforts should be placed on identifying barriers, allocating resources, and building capacity for HPSR.

## Endnotes

^a^Please contact the corresponding authors for the full search strategy.

## Abbreviations

CCS: Country Cooperation Strategies; HIT: Health information technology; HPSR: Health policy and systems research; LMIC: Low- and middle-income countries; WHO: World Health Organization.

## Competing interests

The authors declare that they have no competing interests.

## Authors’ contributions

JX: Drafted the article, conducted the review needed to write article. BM: Assisted in drafting the article and in the review process. NT: Reviewed and edited drafts. AG: Conceptualized the review, reviewed, and edited drafts. All authors read and approved the final manuscript.

## Authors’ information

AG is the Executive Director at the Alliance for Health Policy and Health Systems Research at WHO and has more than 30 years of experience in designing, managing, and evaluating national health systems. NT is the Manager of the Implementation Research Platform at the Alliance for Health Policy and Health Systems Research at WHO. NT also worked as a health systems researcher at Johns Hopkins University and worked with the U.S. Department of Health & Human Services. JX is a pediatric resident physician at the Massachusetts General Hospital, and BM is a preventive medicine resident physician at the University of North Carolina School Medicine. Both JX and BM studied at the Harvard School of Public Health and interned with the Alliance for Health Policy and Health Systems Research at WHO.
